# Evaluating the spatial distribution of *Leishmania* parasites in Colombia from clinical samples and human isolates (1999 to 2016)

**DOI:** 10.1371/journal.pone.0214124

**Published:** 2019-03-27

**Authors:** Clemencia Ovalle-Bracho, Diana Londoño-Barbosa, Jussep Salgado-Almario, Camila González

**Affiliations:** 1 Hospital Universitario Centro Dermatológico Federico Lleras Acosta–E.S.E., Bogotá, Colombia; 2 Centro de Investigaciones en Microbiología y Parasitología Tropical (CIMPAT), Facultad de Ciencias, Universidad de los Andes, Bogotá, Colombia; Universidade Federal da Bahia, BRAZIL

## Abstract

In Colombia, nine species of parasites of the genus *Leishmania* circulate in more than 20 sand fly species, putting at risk of contracting the disease approximately 60% of the population. The Federico Lleras Acosta Dermatological Center, a reference center in Colombia, has been treating patients with cutaneous and mucosal leishmaniasis for more than 15 years, identifying the infecting *Leishmania* species from different clinical samples, and recording systematically all the epidemiological and geographic information related to each diagnosed patient. With this valuable information, the objective of this work was to perform a long term and large-scale study, aiming to identify the *Leishmania* species circulating in Colombia from clinical samples from 1999 to 2016, and to assess their current and potential spatial distribution. In all, four *Leishmania* species were identified in 688 samples from 183 municipalities distributed in 28 of the 32 departments of the country, and 387 records were georeferenced, from 20 departments. The most widespread species was *L*. *(V*.*) braziliensis*, showing new collection records, and the species related to areas with highest leishmaniasis transmission was *L*. *(V*.*) panamensis*. Ecological niche models were built for the three species that had more than 20 georeferenced records, showing a potential distribution for *L*. *(V*.*) braziliensis* on 42% of the national territory mainly in the interandean valleys, and the Orinoquia and Amazon regions. *Leishmania (V*.*) guyanensis* potential distribution covers 36% of Colombia continental territory with a spatial distribution similar to that of *L*. *(V*.*) braziliensis*. There was a marked tendency of *L*. *(V*.*) panamensis* to be distributed in the northwest of the country occupying 35% of the national area and mainly in areas of transformed ecosystems. Species were identified in patients from areas where the occurrence of cases was unprecedented, which suggests that the distribution of *Leishmania* may be greater than currently known. To improve the predictive capacity of the models, we suggest incorporating, in future studies, *Leishmania* samples from vectors and reservoirs that have a greater dependence on environmental variables. Our results are an important tool for health systems because they allow potential areas of transmission and information gaps to be identified.

## Introduction

In the American continent, phylogenetically distinct species of *Leishmania* are known to cause great variation in clinical forms of Leishmaniases [1]. The transmission cycle of this group of diseases is complex, with several *Leishmania* species circulating in different mammal species infected by the bite of Phlebotominae sand flies acting as vectors [2]. Humans are considered as accidental hosts, infected when bitten by a female sand fly in search for a blood source [3].

In Colombia, in 2017, a total of 6,985 cases of Leishmaniases were reported by the National System of Public Health Vigilance (SIVIGILA by its Spanish acronym) (http://portalsivigila.ins.gov.co/sivigila/index.php). From them, 6,870 (98.35%) were of the cutaneous form (CL), 82 (1.17%) of mucocutaneous leishmaniasis (MCL), and 33 (0.47%) of visceral leishmaniasis (VL). These numbers rank Colombia among the top ten countries with highest CL incidence, having 60% of its population at risk of acquiring the disease [4–6]. The country harbors a great diversity of sand fly vectors, and it is estimated that approximately 21 species of them can be involved in parasite transmission in 30 of the 32 departments of the country [3]. Regarding the diversity of parasite species circulating, nine have been reported: six from the *Viannia* subgenus: *Leishmania (V*.*) panamensis*, *Leishmania (V*.*) braziliensis*, *Leishmania (V*.*) guyanensis*, *Leishmania (V*.*) equatoriensis*, *Leishmania (V*.*) lainsoni*, *Leishmania (V*.*) colombiensis* and three from the *Leishmania* subgenus: *Leishmania (L*.*) mexicana*, *Leishmania (L*.*) amazonensis*, and *Leishmania (L*.*) infantum* [7–11]. In Colombia all circulating species have been associated with CL except for *L*. *(L*.*) infantum*, which causes VL. Furthermore, *L*. *(V*.*) panamensis*, *L*. *(V*.*) braziliensis*, and *L*. *(V*.*) guyanensis* have also been related with MCL [12–14].

Although previous studies in Colombia have evaluated sand flies’ current [3,15] and potential distribution, in present and future scenarios [16,17], the spatial distribution of *Leishmania* parasites has not been fully explored. To date, five studies have described nationwide the geographic distribution of *Leishmania*: Corredor et al. (1990) reported the distribution of 340 isolates collected between 1980 and 1988 from 22 departments. The identified species were *L*. *(V*.*) panamensis*, *L*. *(V*.*) braziliensis*, *L*. *(V*.*) guyanensis*, *L*. *(L*.*) amazonensis*, *L*. *(L*.*) mexicana*, and *L*. *(L*.*) chagasi* [7], Ramírez et al. (2016) updated this information up to 2001 and reported two additional species: *L*. *(V*.*) equatoriensis* and *L*. *(V*.*) lainsoni* [10]. Saravia et al. (2002) described the distribution of 1,092 isolates, mainly from the Pacific region, identifying *L*. *(V*.*) panamensis*, *L*. *(V*.*) braziliensis*, and *L*. *(V*.*) guyanensis* [8]. Ovalle et al. (2006) reported species distribution from 137 isolates obtained between 1995 and 2005 from 17 departments [9], and Patino et, al. (2017) reported the distribution of 221 clinical samples from army personnel collected during 2013 in 12 army units located throughout the country [11]. Although the four studies were based on typing and the geographic distribution of *Leishmania* from human isolates was explored, their methodological approaches differed in the number of typed samples, the sample collection period (1980–2017), and the typing method used (i.e. monoclonal antibodies, multilocus enzyme electrophoresis (MLEE), polymerase chain reaction (PCR), and sequencing). Spatial coverage and resolution (natural regions, watersheds, departments, municipalities, or health centers) also differed [7–11].

From these studies, the species most frequently detected in the country has been historically *L*. *(V*.*) panamensis*, followed by *L*. *(V) braziliensis*; less records were found for *L*. *(V*.*) guyanensis* and *L*. *(L*.*) chagasi*, and less than 1% of cases were caused either by *L*. *(L*.*) amazonensis*, *L*. *(L*.*) mexicana L*. *(V*.*) colombiensis*, *L*. *(V*.*) lainsoni*, or *L*. *(V*.*) equatoriensis* [7–11].

Additionally, studies at smaller scales have been conducted, including active search of leishmaniases cases, sand fly collections, and identification of potential reservoirs to pinpoint foci of disease transmission; these studies have allowed to fill in information gaps on the distribution of *Leishmania* [18–20]. However, a long term and large-scale study has not been performed yet.

Knowing the spatial distribution of parasite species with medical importance in areas of disease transmission is relevant because it can contribute to the improvement of the surveillance systems and to the development of new promotion and prevention activities. Ecological niche modeling is a widely used tool, in the context of public health, since it allows to evaluate the potential distribution of species from presence records and environmental variables [21,22]. Through the use of these models, an approach to the ecology of disease transmission can be made, and potential areas of parasite distribution can be identified. Knowing potential areas of disease transmission can facilitate the detection of populations at risk, and the identification of priority areas to design prevention strategies [23,24]. In this context, the objective of this work was to perform a long term and large-scale study, aiming to identify the *Leishmania* species circulating in Colombia from clinical samples obtained by Federico Lleras Acosta Dermatological Center from 1999 to 2016. Spatial distribution of *Leishmania* species was related to areas of disease transmission and their current and potential distributions were evaluated using ecological niche modeling. Description of parasite species distribution in an ecological context, and their relation to potential vector species was also addressed.

## Materials and methods

### Ethical considerations

This study was approved by the Federico Lleras Acosta Dermatological Center Ethics Committee in accordance with the National regulations and the Declaration of Helsinki. The samples included were anonymized to ensure patient confidentiality.

### Clinical samples and *Leishmania spp*. identification

The study included 688 samples obtained from 1999 to 2016, from patients treated at the Federico Lleras Acosta Dermatological Center, Bogotá (Colombia). Of them, 641 had diagnosis of CL and 47 of MCL. The samples included 431 isolates, 76 aspirates, 52 biopsies, and 129 direct smears.

To infer the locality of infection, two surveys were applied to each patient, one during the medical consultation and another during sample collection. Samples linked to inconsistent data between surveys were excluded.

*Leishmania* species were identified by MLEE, PCR followed by restriction fragment length polymorphism (RFLP) of *mini*-*exon* and *hsp70* genes, and sequencing of the amplified products. For typing with MLEE, the enzymes phosphoglucose mutase (PGM, EC 2.7.5.1), glucose-6-phosphate dehydrogenase (G6PDH, EC 1.1.1.49), phosphogluconate dehydrogenase (6PGDH, EC1.1.1.44), nucleoside hydrolase (NH, EC3.2.2.2), superoxide dismutase (SOD, EC1.15.1.1), glucose phosphate isomerase (EC5.3.1.9), and mannose phosphate isomerase (MPI, EC5.3.1.8) were used according to the protocol reported by Saravia et al. [25]. PCR-RFLP was performed for molecular targets *mini*-*exon* and *hsp70*. Amplification products were digested using restriction enzymes *HaeIII* and *BccI*, respectively, following the protocols reported by Ovalle et al. and Montalvo et al. [26,27]; BigDye Terminator v3.1 Cycle Sequencing Kit was used to sequence amplification products selected (Applied Biosystems, Foster City, CA, USA). The results were analyzed in Geneious Pro software v5.5.6. Alignments were performed with reference sequences of the genes of interest reported in NCBI databases. All the obtained records were filed including processing information (typing method), epidemiological (clinical manifestation, sample type) and spatial information (department, municipality, locality, latitude, longitude).

### Spatial distribution

Of the 688 samples for which *Leishmania* species could be identified, those that had information at the locality level were used for georeferencing. Depuration of the geographic database was performed by verifying the existence of the locality in the Geographic Dictionary of Colombia (http://www.igac.gov.co/digeo/app/index.html) and the official web pages of each municipality. Coordinates were assigned using Google Earth and Global gazetteer version 2.3 (http://www.fallingrain.com/world/index.html). The spatial distribution maps of the *Leishmania* species were generated with ArcGIS 10.5.

### Association with transmission areas

To evaluate the distribution of *Leishmania* species in areas where cases have been recorded, the average case rate per 100,000 inhabitants at the municipality level was calculated. Cases of Leishmaniases at the municipality level were only available from 2007 to 2016 and were obtained from SIVIGILA; the population data of the 2005 National Census projected for 2007 to 2016 was gathered from the National Administrative Department of Statistics (https://www.dane.gov.co/index.php/en/). Only *Leishmania* isolates from the same period (2007–2016) were used.

### Potential distribution and ecological characterization

Species with more than 10 occurrences at the locality level (*L*. *(V*.*) panamensis* (n = 229), *L*. *(V*.*) braziliensis* (n = 136) and *L*. *(V*.*) guyanensis* (n = 21) were used to perform analysis of potential distribution through ecological niche modeling (ENM). Due to the enormous self-correlation caused by the number of points present in the same geographic area for *L*. *(V*.*) braziliensis*, *L*. *(V*.*) panamensis* and *L*. *(V*.*) guyanensis* records were reduced using the "*thin*" function of the package "*spThin*" included in the statistical software R [28]. This function allowed the elimination of points that were less than 10 km away, leaving as many points as possible with a total of 76 for *L*. *(V*.*) braziliensis* and 91 for *L*. *(V*.*) panamensis*. The remaining points were retained as a separate dataset for model validation (60 for *L*. *(V*.*) braziliensis*, 6 for *L*. *(V*.*) guyanensis*, and 138 for *L*. *(V*.*) panamensis*).

The calibration area was set as the continental territory of Colombia and 19 derived bioclimatic variables from WorldClim (http://www.worldclim.org/bioclim) at ~1 km resolution, and 45 biweekly layers (2011–2013) at 500m resolution from NDVI (normalized difference vegetation index)Version 5, generated by the Moderate-Resolution Imaging Spectroradiometer (MODIS) were used as the environmental variables. To reduce autocorrelation in environmental variables, principal component analyses (PCA) were performed for each set of variables using ArcMap 10.5. For the bioclimatic variables, the first five PCA accumulated 99.5% of information and for the NDVI the first 11 PCA were required to account for 95% of the variation [29]. To reduce the number of components in the model, initial models were run, using the *MaxEnt* open access program version 3.3.3 (http://www.cs.princeton.edu/~schapire/maxent/), sixteen environmental variables (11 PCA from NDVI, 5 PCA from bioclimatic variables), and three topographic variables (slope, aspect, and topo index) (https://lta.cr.usgs.gov/HYDRO1KReadMe). Conditions of the initial models were 25% test data, bootstrap, two replicates and 1,000 iterations. *Jackknife* analyzes were performed and components contributing the most to the models were selected to run the definitive models [29]. The final models were run using PCA 1, 2, 4 and 5 from the bioclimatic variables, PCA 1, 3, and 7 from NDVI, and as the topographic variables slope, topoindex and aspect. Conditions were set as random seed, bootstrap, 10 replicates, 10,000 iterations and 25% test percentage.

Model validation was performed with two sets of test points: *Leishmania* occurrences not included in the models, and data on vector species published by Ferro et al 2015, considering all sand flies with medical importance as one, due to the non-specific vector-parasite associations. The use of independent evaluation samples is a critical step in model validation [29] thus the use of vector collection data points can be a good alternative for model validation in vector borne diseases. The partial ROC metric was used [30] with E = 10%, 200 simulations, α = 0.05 and p-value 0.05. This method allows to better assess the relationship between omission error for independent points, and the proportion of suitable area in conditions of low omission error [31]. Finally, binary maps were built using the median of the 10 bootstrap replicates and to obtain presence-absence areas.

### Coincidence with potential vector species

In order to establish the coincidence between the potential distribution of *Leishmania* species and sand fly species which could be their potential vectors, the presence areas predicted by MaxEnt and transformed to binary presence/absence layers were overlapped with the records of each insect species of medical importance published by Ferro et al. 2015 [3,32].

## Results

### Clinical samples and *Leishmania* identification

In all, four *Leishmania* species were identified from the 688 isolates, three from the *Viannia* subgenus: *L*. *(V*.*) panamensis* (52.9%), *L*. *(V*.*) braziliensis* (40.7%), and *L*. *(V*.*) guyanensis* (4.8%), and only *L*. *(L*.*) amazonensis* (1.6%) from the *Leishmania* subgenus. *Leishmania (V*.*) panamensis* was identified in the majority of isolates (64.0%), while in biopsies (86.5%) and aspirates (68.4%) *L*. *(V*.*) braziliensis* was the most common species. In the smears, *L*. *(V*.*) panamensis* and *L*. *(V*.*) braziliensis* were identified with equal frequency (45.7%) (Table 1).

**Table 1:** Identification of *Leishmania spp*. by sample type. *Leishmania* species are listed by sample type. Number of records and its equivalent percentage is shown.

Sample type	*Leishmania* species—n (%)				Total
	*L*. *(V*.*) panamensis*	*L*. *(V*.*) braziliensis*	*L*. *(V*.*) guyanensis*	*L*. *(L*.*) amazonensis*	
**Isolate**	276 (64,0%)	124 (28,8%)	21 (4,9%)	10 (2,3%)	431
**Direct smear**	59 (45,7%)	59 (45,7%)	11 (8,5%)	0	129
**Aspirate**	22 (28,9%)	52 (68,4%)	1 (1,3%)	1 (1,3%)	76
**Biopsy**	7 (13,5%)	45 (86,5%)	0	0	52

*Leishmania* species identity was confirmed using at least two methods: MLEE and PCR-RFLP or PCR-RFLP and sequencing. All non-concordant isolates were excluded from the study. MLEE was the method that allowed to identify all the species. PCR of the *mini-exon* gene allowed the identification of samples belonging to *L*. *(L*.*) amazonensis* and the Viannia subgenus, which were posteriorly digested with *HaeIII* to identify *L*. *(V*.*) braziliensis*. Additionally, *L*. *(V*.*) guyanensis* and *L*. *(V*.*) panamensis* were identified by the amplification of the *hsp70* gene followed by *BccI* enzymatic restriction.

Most diagnosed cases of CL (56.3%) were found to be caused by *L*. *(V*.*) panamensis*, followed by *L*. *(V*.*) braziliensis* (36.8%), *L*. *(V*.*) guyanensis* (5.1%), and *L*. *(L*.*) amazonensis* (1.7%). The MCL cases were mostly caused by *L*. *(V*.*) braziliensis* (93.6%) but *L*. *(V*.*) panamensis* was detected in a lower number of cases (6.4%).

### Spatial distribution

According to the geographic origin of the 688 patients from which samples were obtained, *L*. *(V*.*) braziliensis* was the most widespread species, being identified in samples from 106 municipalities in 23 departments of the country, including a new record from the department of Bolívar (municipality of Magangué), where *L*. *(V*.*) braziliensis* had not been reported before. *Leishmania (V*.*) panamensis* also showed a wide distribution in 107 municipalities of 19 departments, followed by *L*. *(V*.*) guyanensis* in 21 municipalities of 9 departments, which also appears as a new record in the department of Huila (municipality of Neiva). Regarding the subgenus *Leishmania*, *L*. *(L*.*) amazonensis* was identified in 11 municipalities of eight departments, of which five had no previous records of this species: Caquetá, Casanare, Cauca, Cundinamarca, and Guaviare (S1 Fig).

**Fig 1:**
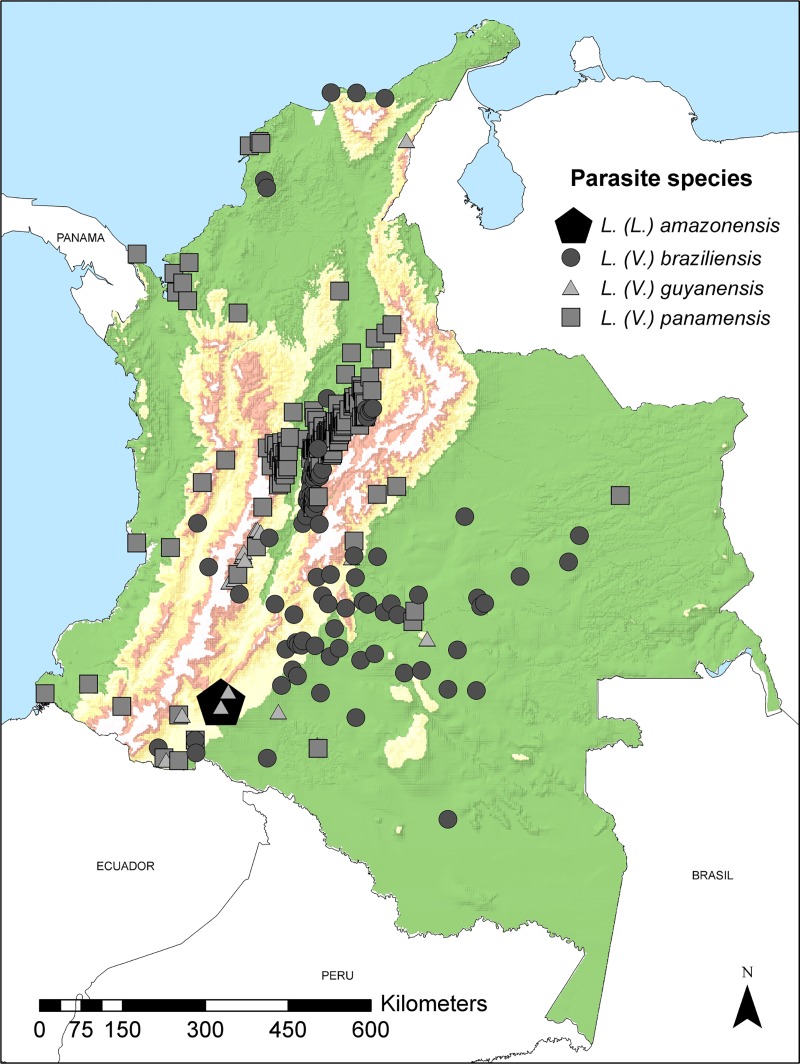
Spatial distribution of *Leishmania* species obtained from human isolates. Localities from human samples were obtained and georeferenced; all records are shown for each typified *Leishmania* species: *L*. *(V*.*) panamensis*, *L*. *(V*.*) braziliensis*, *L*. *(V*.*) guyanensis* and *L*. *(L*.*) amazonensis* in Colombia.

From these records, 387 were georeferenced considering single localities per species in 20 out of the 32 departments of the country: 229 belonged to *L*. *(V*.*) panamensis*, 136 to *L*. *(V*.*) braziliensis*, 21 to *L*. *(V*.*) guyanensis*, and only one record to *L*. *(V*.*) amazonensis* (Fig 1). The majority of records (90%) were distributed at low altitudes between 0 to 1,500 m.a.s.l with the highest proportion (42.3%) occurring between 500 and 1,000 m.a.s.l.

*Leishmania (V*.*) panamensis* was distributed in 16 departments, with 78% of the collection records located in the Natural Andean Region. The localities were mainly distributed in low areas, in the Magdalena River Valley, and 50% of the collection records were in the elevation range from 500 to 1,000 m.a.s.l. Santander had the greatest number of records (n = 67), followed by Cundinamarca (n = 54), Boyacá (n = 38) and Tolima (n = 24).

*Leishmania (V*.*) braziliensis* was identified circulating in 13 departments, with the highest numbers of records obtained in Cundinamarca (36%), Meta (23%), and Caquetá (12%). The records were mainly distributed in municipalities located at low altitudes from 0 to 500 m.a.s.l (44% of the collection records) and between 500 and 1,000 m.a.s.l (28%). This species was distributed mainly in the Orinoquia and Amazon regions towards the east of the eastern mountain range, but it also had records in the Andean region and in the departments of Bolívar, Magdalena, and La Guajira in the Caribbean region.

The 21 records of *L*. *(V*.*) guyanensis* were distributed in seven departments in the center and southwest of the country. The highest number of records was obtained for Tolima (47.6%) together with Boyacá (9.5%) both departments located in the Andean region. The parasite was also isolated in the Caribbean Coast in La Guajira (4.7%) out of the native range of distribution of this parasite. A high number of records was from the Amazon region in Caquetá (19%), Putumayo (9.5%), Guaviare (4.7%) and Meta (4.7%). There was only one record of *L*. *(L*.*) amazonensis*, which was located in the department of Caquetá, in the Amazon region (Fig 1).

### Association with transmission areas

**Fig 2:**
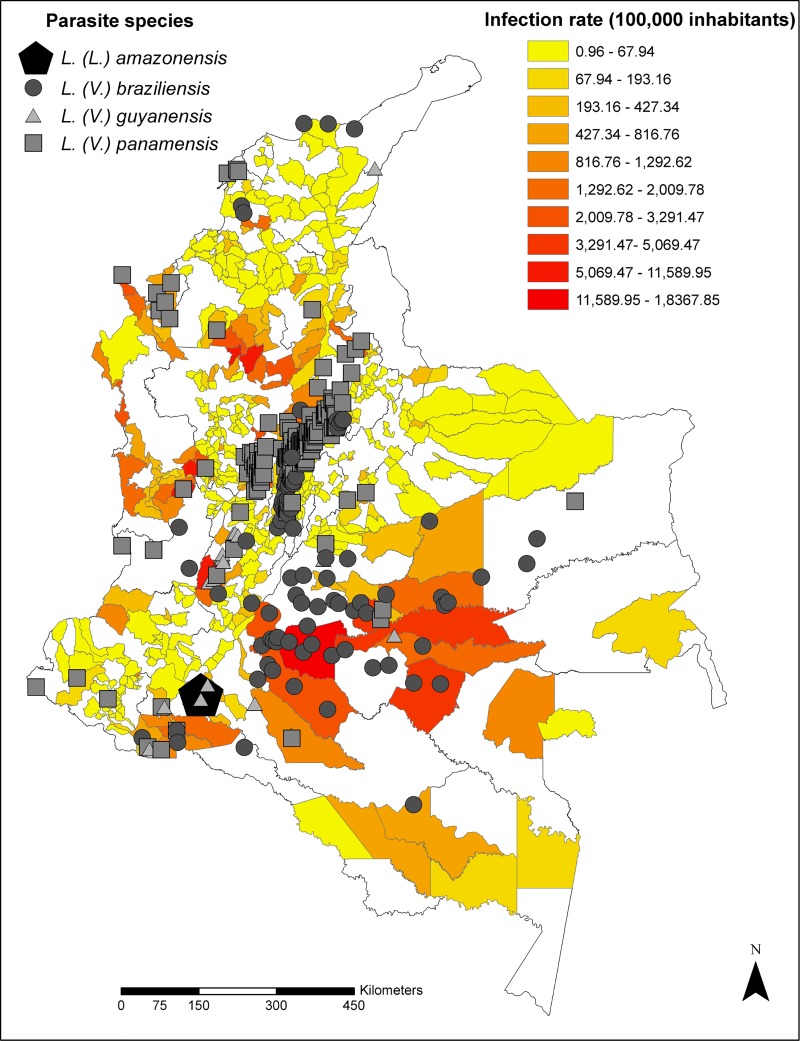
Coincidence between *Leishmania* species distribution and areas with reported cases of leishmaniases. Overlapping of *Leishmania* species distribution obtained from clinical samples and human isolates and areas with cases of Leishmaniases are shown.

In the municipalities with the highest prevalence, generally the species isolated from human samples was *L*. *(V*.*) braziliensis*; however, this species circulated mainly in areas with low prevalence (0.96–67.9 cases per 100,000 inhabitants), as occurred also with *L*. *(V*.*) guyanensis*. On the contrary, *Leishmania (V*.*) panamensis* circulated mainly in areas with medium to high prevalence (Table 2, Fig 2). The municipality with the highest recorded prevalence (18,368 cases per 100,000 inhabitants) was La Macarena in Meta, were *L*. *(V*.*) braziliensis* was identified. Second was Rioblanco, in the department of Tolima, (929 cases per 100,000 inhabitants), where patients infected with *L*. *(V*.*) panamensis* and *L*. *(V*.*) guyanensis* were detected. The third place was San José del Palmar in the Department of Chocó, where *L*. *(V*.*) panamensis* was isolated.

**Table 2:** Distribution of *Leishmania spp*. in areas of leishmaniases transmission. Distribution of *Leishmania spp*. isolates in municipalities where leishmaniases cases have been recorded. Infection rates by 100,000 inhabitants were calculated from 2007–2016.

Cases per 100,000 inhabitants Natural Breaks (Jenks)		*L*. *(V*.*) panamensis*	*%*	*L (V*.*) guyanensis*	*%*	*L*. *(V*.*) braziliensis*	*%*
0.96	67.89	35	24.14	11	57.89	54	49.54
67.89	193.16	2	1.38	1	5.26	5	4.59
193.16	427.34	11	7.59	2	10.53	10	9.17
427.34	816.76	19	13.10	1	5.26	8	7.34
816.76	1,292.62	2	1.38	1	5.26	4	3.67
1,292.62	2,009.79	30	20.69	1	5.26	7	6.42
2,009.79	3,291.47	20	13.79	0	0.00	9	8.26
3,291.47	5,069.47	24	16.55	0	0.00	4	3.67
5,069.47	11,589.96	2	1.38	2	10.53	0	0.00
11,589.96	18,367.85	0	0.00	0	0.00	8	7.34

Records of clinical samples from municipalities for which there was no epidemiological information from the Health National System were found: 28 samples of *L*. *(V*.*) panamensis*, 41 of *L*. *(V*.*) braziliensis*, and nine of *L*. *(V*.*) guyanensis* (Table 2).

### Potential distribution and ecological characterization

Model validation with partial ROC showed greater values of AUC ratio with parasite test points *L*. *(V*.*) braziliensis* (1.77 to 1.91), *L*. *(V*.*) panamensis* (1.84 to 1.94), and *L*. *(V*.*) guyanensis* (1.53 to 1.99), than when validating with sand fly occurrences. These high values reflect that the parasite species dataset used for model validation is not independent from the dataset used for modeling. On the other hand, occurrences of vector species used for testing yielded values above 1 for all parasite species, with AUC ratio values varying from 1.21 to 1.32 in *L*. *(V*.*) panamensis*, from 1.05 to 1.18 in *L*. *(V*.*) guyanensis*, and from 1.12 to 1.26 in *L*. *(V*.*) braziliensis*. These suggest that predictions of parasite spatial distribution are better than random [31].

**Fig 3:**
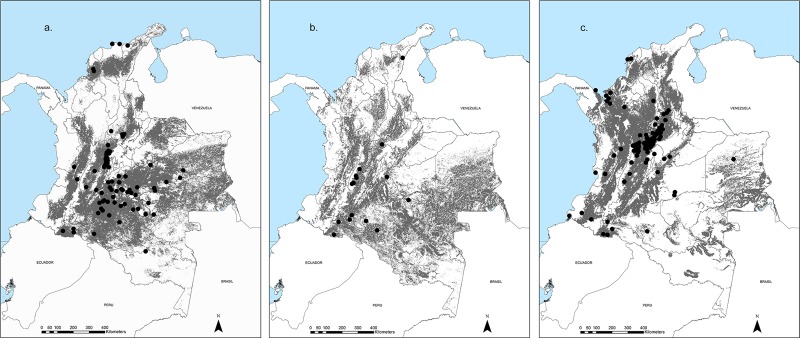
Potential distribution areas for *Leishmania* species. In gray, areas of potential distribution for each parasite species below 2500 m.a.s.l, black dots represent points used to run the models a) *L*. *(V*.*) braziliensis*, b) *L*. *(V*.*) guyanensis* and c) *L*. *(V*.*) panamensis*.

In general, *Leishmania spp* parasites can be distributed in all the continental territory of Colombia. *Leishmania (V*.*) braziliensis* potential distribution covers 42% of the national territory (386,219 Km^2^) and was mainly predicted in the inter-Andean valleys and the Orinoquia and Amazon region. *Leishmania (V*.*) panamensis* has a predominant potential distribution towards the northwestern part of the country, mainly in the Middle Magdalena Valley, the Gulf of Urabá, and the Pacific Coast, and its area of potential distribution covers 35% of the country surface (325,882 Km^2^). *Leishmania (V*.*) guyanensis* is predicted as present in 36% of the national territory (333,968 Km^2^) with a distribution similar to *Leishmania (V*.*) braziliensis*. To refine the areas of potential distribution, predicted areas over 2,500 m.a.s.l were discarded since that is the recorded limit of the elevation range for vector species. The suitable area of distribution for *L*. *(V*.*) braziliensis* is of 375,339 Km^2^ (Fig 3A), for *L*. *(V*.*) guyanensis* 313,572 Km^2^ (Fig 3B), and for *L*. *(V*.*) panamensis* 313,292 Km^2^ (Fig 3C).

Regarding the coincidence with vegetal coverage, all species have similar distribution being predicted mostly in Moist Forest ecoregion (45 to 49%) followed by Montane Forest (19–23%) and Llanos (13%) [33]. Additionally, 57% of the areas of potential distribution of *L*. *(V*.*) panamensis*, 34% of *L*. *(V*.*) braziliensis* and 23% of *L*. *(V) guyanensis*, coincide with transformed ecosystems [34].

### Coincidence with potential vector species

In general, spatial coincidence was observed for known vector species and *Leishmania spp*. For instance, 64% of *Pintomya longiflocosa* ocurrences fall in areas where *L*. *(V*.*) guyanensis* is predicted as present. Other species coinciding with this parasite are *Pintomyia spinicrassa* and *Pintomyia columbiana*. In the case of *L*. *(V*.*) panamensis*, the highest overlap occurs with *P*. *longiflocosa* (88%), *Lutzomyia hartmanni* (86%), *P*. *columbiana* (81%), and, *Psychodopygus carrerai thula* (80%).

*Leishmania (V*.*) braziliensis* was mainly associated to *P*. *columbiana* (85%), followed by *P*. *youngi* (82%), *P*. *spinicrassa* (74%) and *P*. *nuneztovari* (73%) (Table 3).

**Table 3:** Overlapping between potential distribution of parasites and vector species. Percent of localities of each sand fly species falling in the parasite area of potential distribution.

Sand fly species		*L*.*(V*.*) braziliensis*	*L*.*(V*.*) guyanensis*	*L*.*(V*.*) panamensis*
*Psychodopygus*	*amazonensis*	71%	41%	29%
*Nyssomyia*	*antunesi*	58%	46%	28%
*Psychodopygus*	*carrerai carrerai*	71%	46%	33%
*Psychodopygus*	*carrerai thula*	20%	40%	80%
*Pintomyia*	*columbiana*	85%	57%	81%
*Bichromomyia*	*flaviscutellata*	57%	33%	19%
*Lutzomyia*	*gomezi*	52%	35%	66%
*Lutzomyia*	*hartmanni*	50%	50%	86%
*Pintomyia*	*longiflocosa*	67%	64%	88%
*Pintomyia*	*nuneztovari*	73%	55%	73%
*Pintomyia*	*ovallesi*	63%	37%	73%
*Psychodopygus*	*panamensis*	40%	29%	62%
*Psathyromyia*	*shannoni*	45%	22%	55%
*Pintomyia*	*spinicrassa*	74%	68%	37%
*Nyssomyia*	*trapidoi*	35%	35%	72%
*Nyssomyia*	*umbratilis*	50%	33%	33%
*Pintomyia*	*youngi*	82%	45%	82%
*Nyssomyia*	*yuilli*	62%	43%	42%

## Discussion

The present study reunites important information on the spatial distribution of *Leishmania spp*. parasites in Colombia in terms of the volume of data analyzed, their spatial coverage and geographic accuracy, including samples from 688 patients distributed in 28 departments of the 31 that have been identified in the country as endemic for the disease. Different types of samples were used to perform parasite species identification, not only isolates but also clinical samples such as those from direct smears, biopsies and aspirates. The *Leishmania* species most frequently detected belonged to the subgenus Viannia: *L*. *(V*.*) panamensis*, *L*. *(V*.*) braziliensis*, and *L*. *(V*.*) guyanensis*, coincident with previous studies [7]. From the remaining species of the *Viannia* subgenus reported in Colombia (*L*. *(V*.*) equatoriensis*, *L*. *(V*.*) lainsoni* and *L*. *(V*.*) colombiensis*) no samples were found. According to the Colombian National Institute of Health (INS), *L*. *(V*.*) equatoriensis* has been isolated from two patients in the municipalities of Carepa and Yalí, in the department of Antioquia [10]. Our study included samples from Carepa but they were identified as *L*. *(V*.*) panamensis*, showing that several species of the subgenus *Viannia* circulate in this region. On the other hand, *L*. *(V*.*) colombiensis* has been identified in humans in the department of Antioquia [35] and in *Lutzomyia hartmanni* [35] and *Lutzomyia shannoni* [10] sand flies from the department of Santander. Although we had several samples from both departments, we did not find any sample belonging to this parasite.

Regarding the subgenus *Leishmania*, only *L*. *(L*.*) amazonensis* was identified in the analyzed samples. The geographic distribution of other species of this subgenus has been reported in Colombia, and they include more frequently *L*. *(L*.*) chagasi* and also *L*. *(L*.*) mexicana* [7,10]. These works rank *L*. *(L*.*) amazonensis* as the second most frequent species within the subgenus *Leishmania*. The absence of *L*. *(L*.*) chagasi* in this study was mainly due to the fact that this species is related to visceral leishmaniasis cases that are not diagnosed at the Dermatological Center.

It is important to mention that there may be a bias in the recruitment of patients from certain areas because in the department of Antioquia and in Valle del Cauca, there are high-quality reference centers that may be recruiting patients from these regions. For this reason, the greatest concentration of localities of our study came from the Andean and Orinoquia regions. It is important to encourage collaboration and promote data open access in order to better understand the epidemiological scenario of the Leishmaniases in the country.

Regarding the techniques used to perform *Leishmania spp*. identification, in Colombia the most frequent method is the use of isoenzyme patterns and typing with monoclonal antibodies [7,9,36,37]. In recent years, techniques based on the amplification of DNA by PCR have been replacing isoenzyme-patterning techniques and have acquired significant importance in the identification of these parasites, along with the analysis of nucleotide sequences [10,20,38] and RFLP patterns [39]. PCR tests allow parasite species to be identified in some cases based on the size of the amplicon or with additional methods such as RFLP and sequencing [10,20,37,38,40–43]. In this study, parasites from clinical samples like smears, biopsies and aspirates, were typed, thus decreasing the sampling bias associated with parasite culture, which is not always possible to obtain in some clinical forms of the disease.

*Leishmania (V*.*) braziliensis* was the most frequent species found in mucosal leishmaniasis cases. This contrasts with the study conducted by Osorio et al. where *L*. *(V*.*) panamensis* was the predominant species [44]. This can be explained because the patients came from the Pacific region, where this species has a wide distribution. It is important to mention that the present study may also have a bias due to the severity of the clinical forms of the recruited patients, so isolates can belong to species associated with the most aggressive clinical forms that force patients to seek medical attention. Mild or asymptomatic infections may not be detected.

This study allowed to identify species circulating in seven departments and ten municipalities where they had not been reported before, which may suggest that the distribution of the species is broader than known, probably indicating the existence of transmission foci not yet identified by healthcare systems. In general, there was a marked dominance of *L*. *(V*.*) panamensis* in the northwestern region and *L*. *(V*.*) braziliensis* in the southeastern region in both their current and potential areas of distribution. This was possibly due to the presence of suitable vector species of each of the parasite species, which have a distribution delimited by the Andean mountain ranges [3].

For the species, *L*. *(V*.*) guyanensis*, considering that the greatest number of records belong to the department of Tolima, it is clear that it constitutes a species that broke its native range of sylvatic distribution in Colombia, as it was found in 2004 in Chaparral, Tolima, infecting humans and *P*. *longiflocosa* [20]. The current known distribution of the parasite is greater than previously expected, being found in the Caribbean Coast [45]; the ENM for this species confirm its dispersal potential as it founds suitable insect vectors.

Regarding the overlap with transmission areas, it is important to highlight the importance of epidemiological surveillance because some isolates were obtained from municipalities for which there were no records of cases in the period between 2007 and 2016. There is a significant underreporting of leishmaniases cases not only in Colombia but also in other endemic regions in the world. Therefore, it is possible that cases are occurring in localities that were not reported to the health system. Additionally, the possibility of errors in the spatial location of parasites should not be ruled out because patients are omitting or giving inaccurate information about the probable place of infection because it is a disease that has been largely associated with armed groups [40,46].

Although the georeferencing process was reliable, and we have an acceptable number of samples to perform spatial distribution modeling, it is important to bear in mind that humans are being used as a proxy to build an ecological niche for the parasite. We are aware that there are several methodologic approaches that can be used to reach the optimal level of model complexity, such as selecting the features classes and regularization coefficients in MaxEnt. However, the data we are using in this study imposes the challenge of modeling parasitic species isolated from the most ubicuous host in the planet (humans), which are not necessarily highly dependent on environmental variables. For further studies on *Leishmania* potential distribution, we strongly suggest to use parasite samples isolated from vectors and reservoirs that have a narrower dependence on environmental variables, and perhaps use humans and non-infected specimens as an independent set to evaluate the models [41, 47].

Relating the areas of potential distribution of parasites and vector’s occurrences, a coincidence was observed between *L*. *(V*.*) guyanensis* and its proven vector *Pintomyia longiflocosa*. Ferro *et al*. found this species naturally infected with *L*. *(V*.*) guyanensis* in Chaparral, Tolima, in the Andean region [20]. This sand fly species has also been related with *L*. *(V*.*) guyanensis* transmission due to its high abundances in the upper and middle Magdalena River valley (sub-Andean region) during leishmaniases outbreaks [3,20,48]. Other sand fly species coinciding with *L*. *(V*.*) guyanensis* are *P*. *spinicrassa*, *P*. *columbiana* and *L*. *hartmanni* (also for *L*. *(V*.*) panamensis*) however, this association has not been found occurring in nature.

*Leishmania (V*.*) panamensis* was found mainly coinciding with the presence of *P*. *longiflocosa* and *P*. *columbiana*, but these sand flies have not been related so far to transmission of this parasite. Other important coincidences were found with *L*. *gomezi* and *N*. *trapidoi*. This parasite has been detected naturally infecting *L*.*gomezi*, and *P*. *panamensis* in Córdoba [45], and Pauna, Boyacá [49]; *N*. *trapidoi* in Arboledas, Santander [50] and in Tumaco, Nariño [51]; and *Nyssomyia yuilli* in Otanche, Boyacá [49].

*Leishmania (V*.*) braziliensis* also overlaps mainly with *P*. *columbiana*; Bejarano et al found this species along with *P*. *panamensis* and *P*. *evansi*, in the urban area of Sincelejo, Sucre, and suggested that these species could be related with the transmission of *Leishmania (V*.*) braziliensis* [52]. Other species with high coincidence are *P*. *youngi*, *P*. *carrerai carrerai*, *P*. *longiflocosa*, *P*. *nuneztovari*, and *P*. *ovallesi*. Young et al. found promastigotes inside the gut of *P*. *spinicrassa* sand flies in Arboledas, Norte de Santander [50], while Warburg *et al*., reported artificial infection in wild-caught sandflies from five sand fly species collected in four municipalities in Valle del Cauca. They found that *L*. *townsendi* and *L*. *lichyi* showed the highest percentages of infection followed by *P*. *columbiana* and *L*. *pia* [53]. Pardo *et al*. suggested *P*. *longiflocosa* and *P*. *columbiana* as potential vectors for *L*. *(V*.*) braziliensis*, considering their dominance and endophagia in three leishmaniases outbreaks in the sub-Andean region of Colombia: Huila, Planadas (Tolima) and Abrego (Norte de Santander) [48]. *Pintomyia nuneztovari* and *P*. *ovallesi*, have been infected artificially with promastigotes, and were found in an outbreak in Reventones, Cundinamarca [54].

The species *P*. *carrerai carrerai* has not been incriminated as vector of any *L*. *(V*.*) guyanensis* nor *L*. *(V*.*) braziliensis* in Colombia, however, this species has been related with infection of *L*. *(V*.*) braziliensis* in Brazil [55] and Bolivia [56]. Likewise, *N*. *yuilli* was only found as vector of *Leishmania spp*. in Perú and *P*. *youngi* as potential vector of *L*. *(V*.*) braziliensis* in Venezuela but there is no information about natural infection of this species with *L*. *(V*.*) braziliensis* or *L*. *(V*.*) guyanensis* in Colombia [57].

In general terms, there are competent vector species related to areas of *Leishmania spp*. potential distribution, and this has important implications for the establishment of new transmission foci. It is important to emphasize that the potential distribution of parasites based on human isolates must be carefully considered and used as a tool to identify potential areas of transmission, rather than as a spatial delimitation of the distribution of the species. Strictly, the distribution of *Leishmania spp*. will depend on the presence of insect vectors and there is an enormous potential for vector species to change their spatial distribution, especially under climate and land use changes that are being recorded on a global scale [16,17,58]. For this reason, we encourage the health systems to perform adequate vector surveillance and carefully monitor areas where the presence of vector insects is known, to establish adequate prevention strategies.

## Supporting information

S1 Fig Administrative division of Colombia showing municipalities with new records for each *Leishmania* species. (JPG)
