# Supplementary figures and images for: Evaluating the spatial distribution of Leishmania parasites in Colombia from clinical samples and human isolates (1999 to 2016)
Source: PLoS One. 2019 Mar 27;14(3):e0214124. doi: 10.1371/journal.pone.0214124 (PMC6436702; doi:10.1371/journal.pone.0214124)

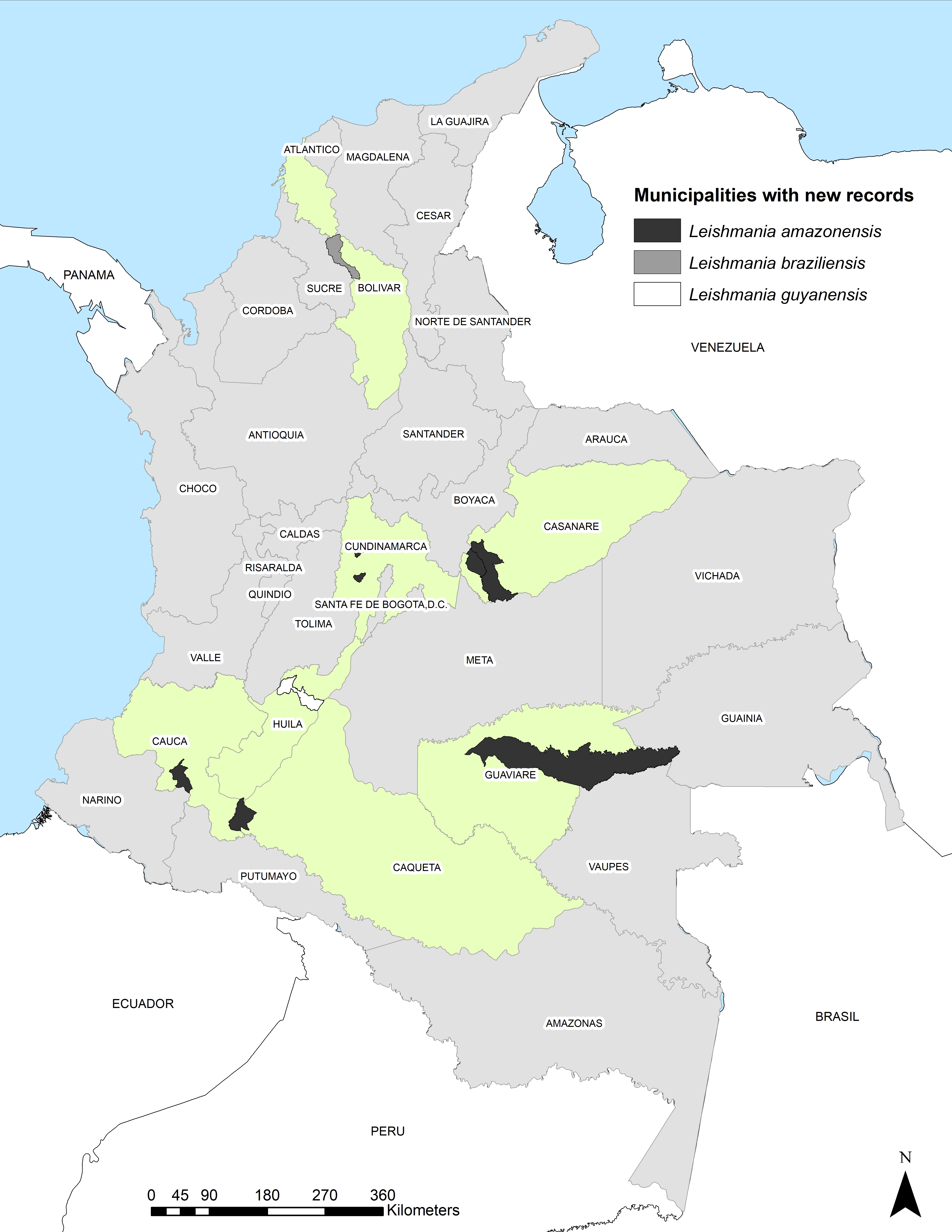

Supplement: S1 Fig — (JPG) [file pone.0214124.s001.jpg]
